# Development of Dynamic Four-Dimensional Printing Technology for Patterned Structures by Applying Microcellular Foaming Process

**DOI:** 10.3390/polym16162242

**Published:** 2024-08-07

**Authors:** Kwan Hoon Kim, Jae Hoo Kim, Jin Hong, Sung Woon Cha

**Affiliations:** 1 School of Mechanical Engineering, Yonsei University, 50 Yonsei-ro, Seodaemoon-gu, Seoul 03722, Republic of Korea; kimkevin99@yonsei.ac.kr (K.H.K.); jin.hong@yonsei.ac.kr (J.H.); 2 Convergence Research Center for Solutions to Electromagnetic Interference in Future-Mobility, Korea Institute of Science and Technology (KIST), 5, Hwarang-ro 14-gil, Seongbuk-gu, Seoul 02792, Republic of Korea; murja@kist.re.kr

**Keywords:** microcellular foaming process, batch foaming process, polymer, 4D printing, patterning

## Abstract

Four-dimensional (4D) printing adds the dimension of time to 3D-printed specimens, causing movement when external stimuli are applied. This movement enables applications across various fields, including the soft robotics, aerospace, apparel, and automotive industries. Traditionally, 4D printing has utilized special materials such as shape-memory polymers (SMPs) or shape-memory alloys (SMAs) to achieve this movement. This study explores a novel approach to 4D printing by applying microcellular foaming processes (MCPs) to 3D printing. This study primarily aims to design and fabricate patterned specimens using common materials, such as PLA, through 3D printing and to analyze their dynamic behavior under various foaming conditions. To demonstrate the potential applications of this technology, the degree of bending was measured by controlling the patterning and foaming conditions. The 3D-printed specimens with microcellular foaming exhibited predictable deformations owing to the asymmetric expansion caused by differential gas saturation. The results confirm that 4D printing can be realized using conventional materials without the need for smart materials and can introduce foaming processes as a new external stimulus. This study highlights the potential of combining 3D printing with microcellular foaming for advanced 4D printing applications.

## 1. Introduction

Three-dimensional printing was developed to manufacture complex shapes by cutting, which is a traditional manufacturing process [1,2]. Additive manufacturing refers to the process of manufacturing structures and parts by stacking layers individually using metal and polymer materials. Three-dimensional printing has advanced significantly and has become an essential technology in various industries that require high precision, such as aerospace, automobiles, medical devices, and engineering. This technology reduces material waste and enables effective prototyping and production for custom manufacturing, thereby reducing the manufacturing time. Recent works have explored ways to improve the performance of solid oxide fuel cells (SOFCs) by 3D printing conductive polymer-based materials [3].

With the advancement of technology, researchers and engineers have continued to study new methodologies to broaden the limits that can be achieved through 3D printing. One such approach is the development of four-dimensional (4D) printing, which refers to a technology in which a time axis is added to a three-dimensional shape. In other words, 3D-printed objects react to environmental factors, such as temperature, humidity, light, and chemical reactions, and transform themselves under the influence of time. Four-dimensional printing was first used by Dr. Skylar Tibitz at MIT in 2013 [4].

Four-dimensional (4D) printing typically uses smart materials that change shape depending on external environmental factors [5,6,7]. This creates an appearance by 3D printing smart materials and causes movement owing to changes in external factors. Smart materials, including shape-memory alloys (SMAs), shape-memory polymers (SMPs), and materials such as hydrogels and wood, can also be deformed. Four-dimensional printing has opened up various applications in automobiles, robots, medical care, manufacturing, and construction.

An SMP is a substance that maintains a temporary shape and is restored to its memorable shape by external stimulation. Therefore, numerous studies on 4D printing using SMPs are being conducted. SMPs have easy movement control; however, they are expensive compared to general materials and have weak mechanical properties [8,9]. Accordingly, many studies on SMPs and general materials are currently being conducted in 4D printing research.

At MIT, David Correa et al. implemented 4D printing using the anisotropy and hygroscopicity of wood materials [10,11]. Wood materials exhibited expansion and contraction characteristics based on the effect of moisture, and self-deformative structures were produced using this method. Existing studies have implemented movement using smart materials; however, research has confirmed that motion can only be implemented with general materials without using SMPs.

Several 4D printing studies have also been conducted on a material called hydrogel [11,12,13,14]. Hydrogels can absorb water and expand their volume, and some studies have implemented movement through additive directions and patterns using hydrogels. The degree and direction of bending can be controlled according to the printing path and the shape of the pattern. In addition, studies have been conducted to implement movement using bi-materials. This study investigated the occurrence of bending due to the difference in the volume expansion of bi-materials, and bending was controlled by considering the difference in the amount of moisture absorption and the characteristics of the material [12,13].

In addition to research on the properties of materials, studies are also being conducted to fabricate soft actuators using additives. Eristoff et al. produced soft actuators using a solvent called perfluorodecalin (PFD) [14]. PFD changes from liquid to gas at a certain temperature or higher, causing the volume expansion of particles. Movement was observed in a specimen produced using composite PFD on silicon, in which the volume increased owing to the phase change in the grain when heat was applied. In addition, it was confirmed that by patterning via 3D printing, the specimen could move as a one-time actuator [15].

The microcellular foaming process (MCP) refers to the process of forming small and uniform cells of 10 μm or less in the polymer matrix in 10^9^ cells/cm^3^ or more [16,17,18]. The microcellular foaming process comprises two steps: the first is a saturation process in which a polymer material is placed in a pressure vessel and high-pressure gas is injected to saturate the gas, and the second step is the formation of cells in the polymer owing to the thermodynamic instability of a specimen saturated with gas. Thermodynamic instability includes sudden changes in pressure, temperature, and pressure. The MCP expands the volume of the polymer and can control weight reduction, material reduction, increased impact strength, and thermal, acoustic, and electrical properties [19,20,21].

Yang et al. induced bending deformation through light using non-uniform thermal expansion with thermal expandable microscopes (TEMs) and an additive such as a chemical foaming agent [22]. TEMs were added to polydimethylsiloxanes (PDMs) and combined with a single layer and a pure PDMS layer used as active layers to produce the same movement as a bi-material actuator. When heat was applied to the prepared specimen, a chemical reaction occurred in the active layer containing TEMs, volume expansion occurred, and the pure PDMS layer exhibited relatively smaller expansion, resulting in bending owing to non-uniform thermal expansion. In addition, Lim et al. observed bending through foaming using the bilayer structure of a bi-material [23].

Authors of previous studies have conducted extensive experiments on the implementation of 4D printing using SMPs or additives. However, experiments utilizing common materials or proposing new methods for external stimuli are limited. Additionally, experiments controlling bending due to volume expansion typically do not use single materials but rather employ bi-materials. In this study, we attempted to control bending by using foaming through patterning using PLA, a single material belonging to the general materials class. Therefore, bending motion can be achieved by controlling the foaming rate through patterning, owing to the non-uniform volume expansion from patterning. This study presents a novel 4D printing mechanism that combines 3D printing with a microcellular foaming process.

## 2. Materials and Methods

### 2.1. Materials

#### 2.1.1. Specimen

This study used a PLA filament (Shenzhen Esun Industrial Co., Shenzhen, China). PLA is typically the most used resin in 3D printing because it has low shrinkage and is an eco-friendly material made from plants such as corn and sugarcane, making it suitable for 3D printing. Polylactic acid (PLA) materials are semi-crystalline resins with high gas saturation and foaming rates that are widely used in foaming studies [19,24]. The density of the PLA material is 1.24 g/cm^3^, and the glass transition temperature is 52 °C. The conditions used in the actual 3D printing process were to prepare a specimen by setting the conditions at 210 °C for the process temperature and 60 °C for the bed temperature.

#### 2.1.2. Blowing Agents

The MCP is a process in which gas is injected into a molten polymer to change it. It comprises different aspects based on the gas medium. Typical gases include N_2_ and CO_2_, selected based on how the desired properties are controlled. In particular, because the physical properties and internal morphology change depending on the foaming agent used, an appropriate gas should be used. The difference between N_2_ and CO_2_ is that their solubilities and diffusivities differ [25,26,27]. N_2_ gas is used for physical foaming injection molding, which requires rapid solubility and high pressure. In batch foaming, CO_2_ gas, which has high solubility and dissolves easily even at low pressures, is used.

In this study, a batch foaming process was applied to 3D-printed specimens; when a blowing agent with high solubility was used, the foaming rate increased and significant bending occurred [27]. Therefore, CO_2_ was selected owing to its high solubility. In this study, a gas solvent was selected as CO_2_ (99.9% purity, 40 L, Seoul Samheungastec, Seoul, Republic of Korea).

### 2.2. Experiment

#### 2.2.1. Microcellular Foaming Process

The MCP is divided into physical foaming and chemical foaming processes and, in this study, a batch foaming process was used during the physical foaming process [28,29]. The physical foaming process involves saturating gas in a polymer and a foaming process in which cells are formed through thermodynamic instability. Since high-pressure gas must be saturated in the polymer, a pressure vessel is required. The radius and height of the pressure vessel used in this study were 100 mm and 200 mm, respectively. The thermodynamically unstable polymer specimen was heated in an oil bath to perform the foaming process.

#### 2.2.2. Three-Dimensional Printing of Patterned Specimens

**Figure 1:**
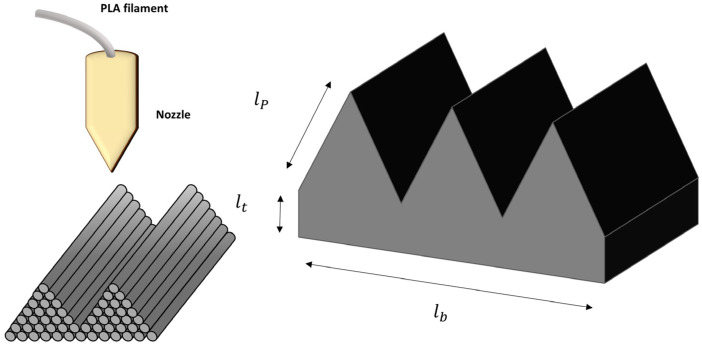
Schematic diagram for 3D printing to pattern the specimen.

The specimens were produced using PLA material and patterned using a 3D printer (DP 103, Shindoriko, Seoul, Republic of Korea). The nozzle temperature was set to 210 °C, and the bed temperature was set to 60 °C according to the process temperature of the 3D printing material. In addition, the size of the nozzle was 0.2 mm, and the layer thickness was set to 0.2 mm. The filling amount and form are factors that affect the bending research and mechanical properties. The detailed 3D printing conditions are listed in Table 1 The pattern of 3D printing was set as shown in Figure 1, and a specimen was produced according to the length of the pattern. The thickness of the patterned surface $l_{t}$ was fixed at 1 mm, the length of the specimen $l_{b}$ was fixed at 60 mm, and the length of the pattern $l_{p}$ was set to 1, 2, and 3 mm. The length was adjusted to measure the degree of bending during foaming according to changes in the length of the pattern.

#### 2.2.3. Microcellular Foaming 4D Printing

**Figure 2:**
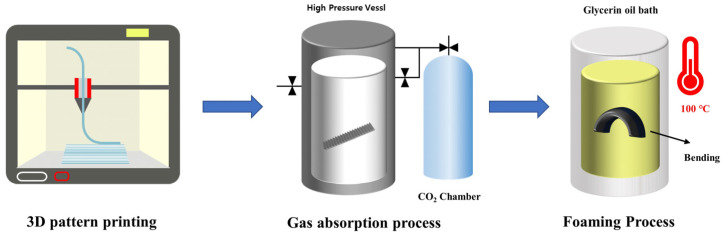
Schematic diagram of microcellular 4D printing.

This study aimed to implement 4D motion by manufacturing a patterned specimen using 3D printing and then applying an MCP. Most existing 4D printing methods implement 4D printing using the characteristics of smart materials. Therefore, these approaches are expensive owing to the price of the material and the low mechanical properties of smart materials. However, microcellular foaming 4D printing produces a patterned specimen using PLA, which is a common material, and a 3D printer. Subsequently, 4D motion is implemented using the formation of cells that appear by applying MCPs. Figure 2 illustrates the process of microcellular 4D printing, visually representing the step-by-step procedure of fabricating patterned specimens using PLA, applying MCP, and subsequently forming cells to achieve 4D motion. Consequently, unlike existing 4D printing methods, a single common material was used, and an MCP was proposed as a new stimulus.

### 2.3. Characterization

**Figure 3:**
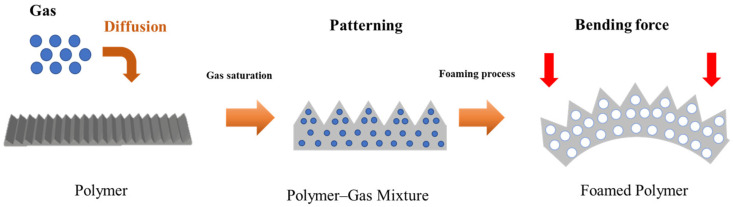
Schematic diagram of patterning in 4D printing.

The MCP initiates volume expansion by forming pores inside the polymer specimen [30,31]. Herein, asymmetric volume expansion occurred in the patterned and non-patterned parts, as shown in Figure 3, and bending occurred accordingly. Therefore, bending is affected by cell nucleation, the foaming rate, and the degree of bending changes. Research has been conducted to control bending based on the MCP parameters [32]. The MCP changes the size of the cells and foaming rate according to parameters such as temperature, pressure, time, compression ratio, and foaming temperature for gas saturation [31]. In this study, the foaming rate according to gas saturation was controlled by controlling the saturation time of the MCP, which affects the foaming rate significantly. The detailed conditions used in the study were tested under the conditions shown in Table 2. Gas saturation was measured using a weight meter (AR2130, OHAS Corp., Parsippany, NJ, USA) with a precision of 0.001 g. The weights of the polymer specimens were measured before and after gas saturation.

The weight gain of the dissolved gas can be determined using Equation (1).

(1) $$\mathrm{Gas}\;\mathrm{sorption}\;\left(\%\right)=\frac{{\mathrm{Weight}}_{\mathrm{after}}\;\left(g\right)-{\mathrm{Weight}}_{\mathrm{before}}\left(g\right)}{{\mathrm{Weight}}_{\mathrm{before}}\;\left(g\right)}$$

Next, the density of the specimen was measured using a density meter (MD-300s, Alpha Mirage, Miyakojima-ku, Japan) according to ASTM D792 to measure the foaming rate. The density of each specimen was measured before and after foaming, and the foaming rate was calculated using Equation (2).

(2) $$\mathrm{Foaming}\;\mathrm{ratio}\;\left(\%\right)=\frac{\rho_{before}\;\left(g/cm^{3}\right)-\rho_{after}\;\left(g/cm^{3}\right)}{\rho_{before}\;\left(g/cm^{3}\right)}$$

**Figure 4:**
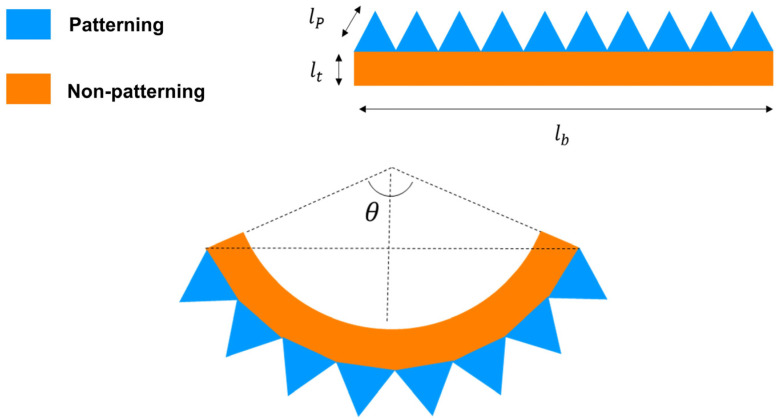
Schematic diagram for bending calculation.

Optical images were used to accurately measure the bending phenomena. Imaging equipment (Galaxy S23 Ultra, Samsung, Gumi, Republic of Korea) was used to capture the optical images. The bending measurement process is described in Figure 4 [22,23,33]. Field-emission scanning electron microscopy (FE-SEM) (JSM-IT500, JEOL, Tokyo, Japan) was performed to confirm the internal cell morphology of the polymer and was used to confirm the effect of cell morphology on the bending behavior. Before the SEM measurement, the fracture specimen was coated with platinum (Pt) for 200 s in a vacuum using a sputter coating device (108 auto sputter coater, Cressington Scientific Instruments, Watford, UK).

## 3. Results and Discussion

### 3.1. Foaming Characteristics

#### 3.1.1. Gas Absorption

**Figure 5:**
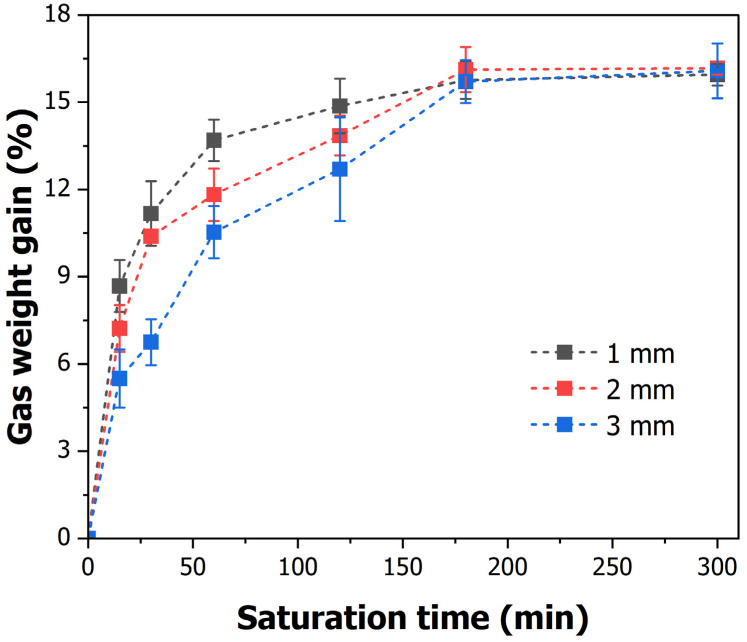
CO_2_ weight gain (%) according to saturation time and pattern size.

The influence of gas pressure, temperature, and time is one of the main parameters in gas saturation; however, the material used is also crucial. The gas saturation and foaming rate differ significantly, even under the same conditions, based on the characteristics of the material [34]. Therefore, the change in the gas saturation of the PLA material used in this study was measured, as shown in Figure 5. The saturation conditions for the gas were set as listed in Table 2. The gas saturation increased as the saturation time increased.

In this study, gas saturation changed according to the patterning length, which was set to 1, 2, and 3 mm. The gas saturation increased gradually as the saturation time increased. At 15 min, the 1 mm specimen had a gas weight gain of 8.68%, whereas that of the 2 mm and 3 mm specimens was 7.22% and 5.5%, respectively. The gas saturation was found to vary depending on the patterning length of the specimen. However, the saturation of all the specimens became almost the same at approximately 16% after 180 min, and it was almost the same at 16% even after 5 h. Thus, the maximum gas saturation at 5 MPa of the PLA material was confirmed to be 16%, and the maximum saturation time was 180 min.

#### 3.1.2. Foaming Ratio

**Figure 6:**
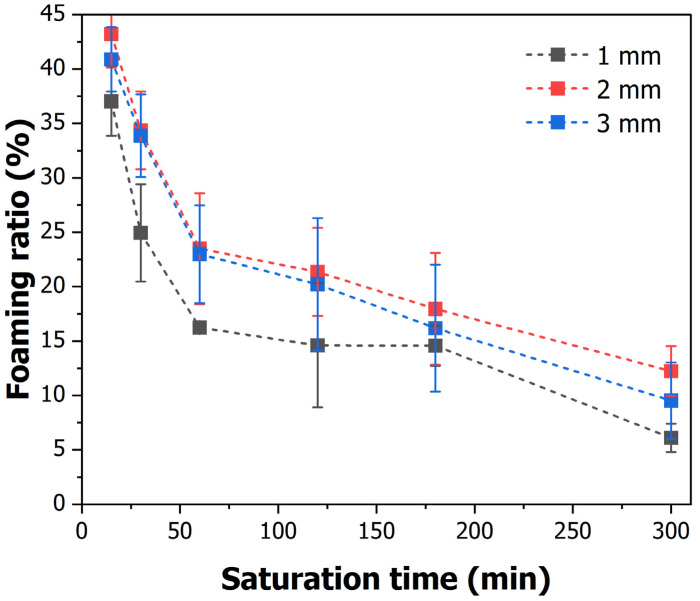
Foaming ratio according to saturation time and pattern size.

As shown in Equation (2), the foaming rate was calculated using the difference between the densities before and after foaming. The conditions for the placement process were set, as shown in Table 2, to confirm the difference in the foaming rate according to the saturation time. In addition, seven specimens were used, and the average was obtained, excluding the maximum and minimum values, to ensure experimental accuracy. The foaming rates measured in this study are shown in Figure 6.

In all the specimens, the gas saturation increased as the saturation time increased, but the foaming rate gradually decreased. The highest foaming rate was observed at 15 min, with rates of 37.03%, 43.22%, and 40.89% observed for patterning lengths of 1, 2, and 3 mm, respectively. In addition, the lowest foaming rate was observed at 5 h, which is the maximum time, with rates of 6.1%, 12.24%, and 9.53% observed for patterning lengths of 1, 2, and 3 mm, respectively. At all times, the highest foaming rates were observed in the 2 mm pattern, and the lowest foaming rate was observed in the 1 mm pattern. The results showed that the foaming rate tended to decrease over time and that the foaming rate differed depending on the pattern.

The differences in gas saturation according to the pattern size are due to the surface area-to-volume ratio. The 1 mm patterned specimens have a higher surface area relative to their volume, resulting in a faster gas absorption rate compared to the larger volume 3 mm patterned specimens. Consequently, since the surface area is the same, the gas absorption rate is highest in the 1 mm specimens and lowest in the largest volume 3 mm specimens.

However, as gas diffusion reaches equilibrium, all specimens exhibit the same maximum gas saturation of 16% after 180 min. This indicates that gas diffusion is influenced by the dimensions, surface area, thickness, and material characteristics of the specimens.

#### 3.1.3. Cell Morphology

**Figure 7:**
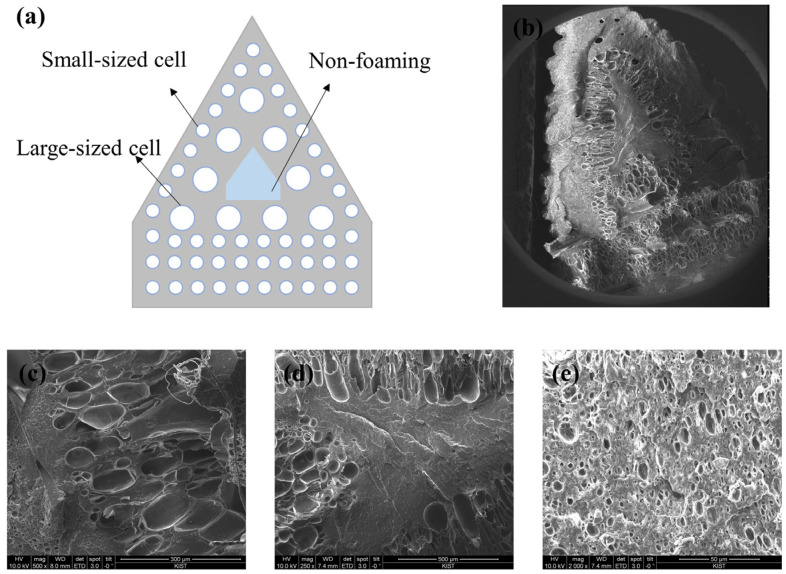
The FE-SEM images of the patterned specimen. (**a**) Schematic diagram of cell morphology. (**b**) SEM image of the overall specimen. (**c**) SEM image of the inner section. (**d**) SEM image of the non-foaming section. (**e**) SEM image of the outer section.

FE-SEM images were observed to investigate the internal structure of the patterning specimen. The internal cell morphology of the specimen is shown in Figure 7. The cell morphology of the specimen changed according to the patterning length and saturation time. The morphology of the cells changed over time, attributable to gas diffusion. Furthermore, cell formation differed between patterned and non-patterned surfaces, leading to asymmetric volume expansion.

**Figure 8:**
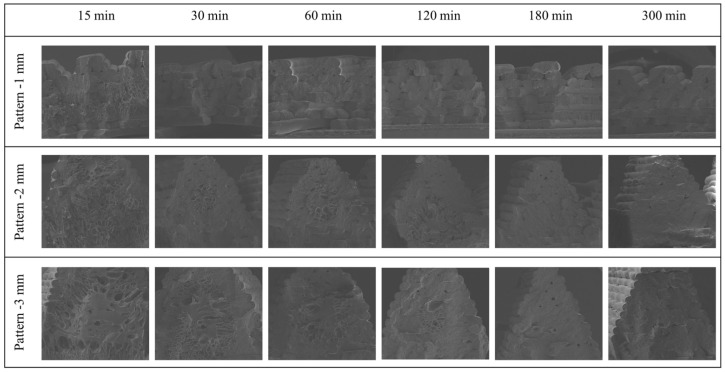
The FE-SEM images of foaming in the patterned specimen according to saturation time and pattern size.

Figure 8 shows that the heterogeneous cell is generated before 120 min and that the morphology of the cell is generated in bimodal form. Small cells were formed outside the cell, as shown in Figure 7e, whereas large cells were placed inside, as shown in Figure 7c. As shown in Figure 7d, at shorter durations, no cells were observed in the center of the pattern. In addition, the gas was completely saturated, and a homogeneous cell was formed after 180 min, thus confirming that the size and density of the cell can be adjusted according to the degree of gas diffusion.

### 3.2. Bending Dynamics Induced by Patterning and Foaming

**Figure 9:**
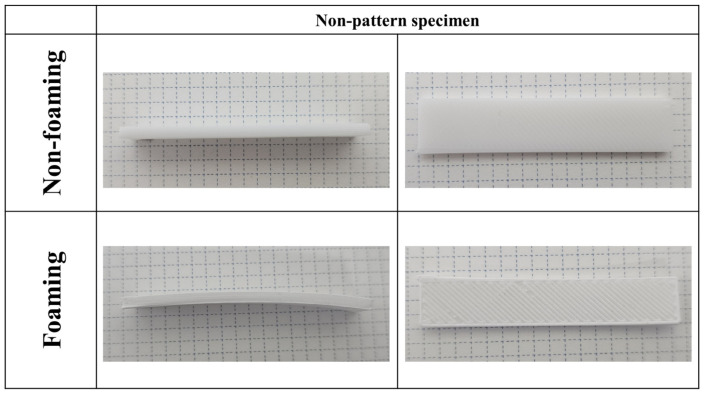
Non-patterned specimen according to the foaming process.

**Figure 10:**
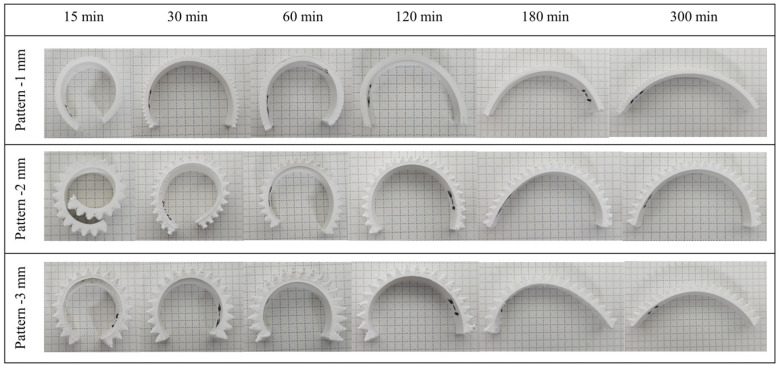
Bending angle according to the saturation time.

Bending did not occur when the foaming process was applied to the non-patterned specimen, as shown in Figure 9; however, bending occurred following the foaming of the patterned specimen. In addition, the degree of bending was confirmed to vary depending on the saturation time of the gas and the patterning length, as shown in Figure 10.

When the gas saturation time was 15 min, approximately 330°, 410°, and 335° of bending occurred in the 1, 2, and 3 mm patterns, respectively. The most bending occurred at 15 min, which had the highest foaming rate. In addition, bending gradually decreased as the saturation time increased, with the 1, 2, and 3mm patterns exhibiting the lowest bending at 125°, 155°, and 129°, respectively.

The degree of bending is caused by the asymmetry of cell formation on the patterned and nonpatterned surfaces, which is related to the foaming rate. The shorter the saturation time, the greater the proportion of large cells, resulting in a large volume expansion in the pattern. In addition, if the saturation time is short, heterogeneous cells are formed, increasing asymmetry. Consequently, the foaming rate was high because of the increase in cells over a short period, and the bending angle was large, as determined by examining the internal cell morphology; that is, when the foaming rate was high, volume expansion occurred, and greater stress due to asymmetry was generated, indicating that the degree of bending was also high.

**Figure 11:**
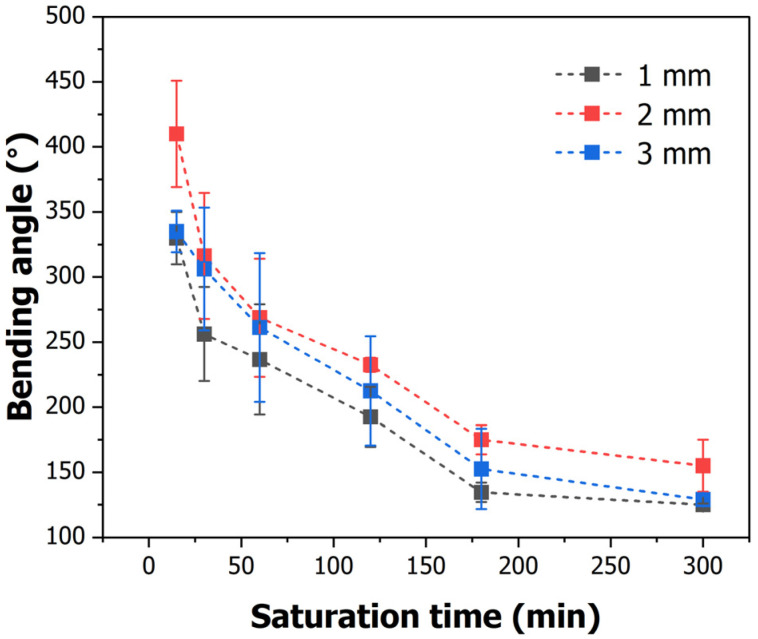
Bending angle according to saturation time and pattern size.

**Figure 12:**
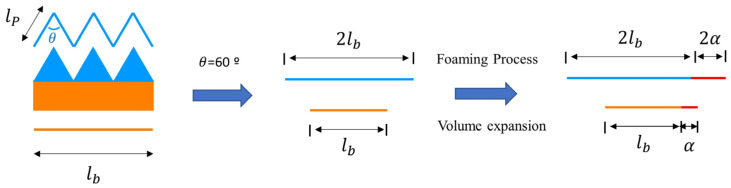
Bending behavior in a homogeneous cell structure.

Additionally, as the saturation time increased and full saturation was achieved, the cell size decreased and the foaming rate decreased, resulting in less bending. After 120 min, when fully saturated, the cells were homogeneously distributed, and the bending at this time is shown in Figure 11. The case where homogeneous cells are generated can be explained as shown in Figure 12. The surface area length $l_{p}$ of the patterned side is twice that of the length $l_{b}$ of the specimen, owing to the angle θ being 60°. If volume expansion due to foaming occurs equally at this time, α, the difference between the strain rates of the patterned and non-patterned surfaces is up to the value α, resulting in bending. Although the length of the pattern is different, if the length of the surface area is the same, the degree of bending of the specimen becomes similar as homogeneous cells are generated after 180 min, as shown in Figure 10.

### 3.3. Application

**Figure 13:**
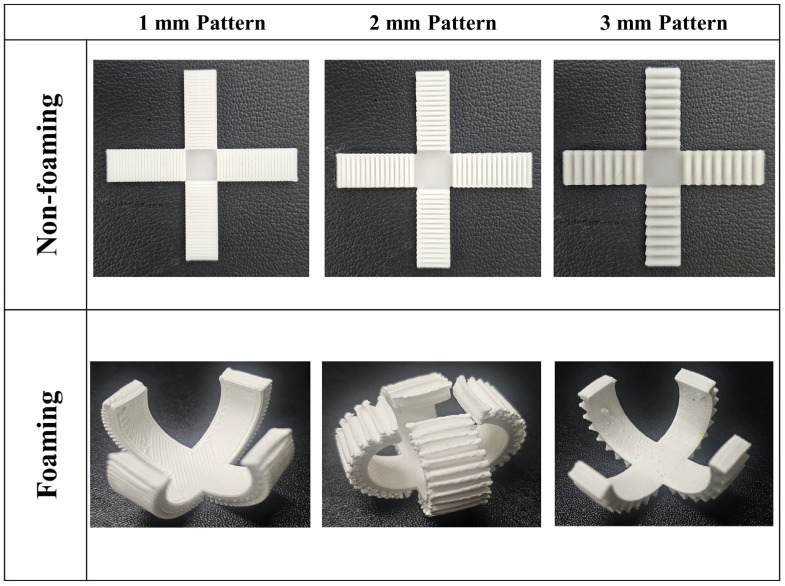
Application of a cross shape.

Through foaming of the patterned specimen, a cross shape, as shown in Figure 13, was implemented using 4D motion, producing a cross shape-patterned specimen, as shown in Figure 13, in which the shape was spontaneously deformed by thermal stimulation. The four sides of cross-bending occurred in a patternless area when the foaming process was applied. This bending could be adjusted according to the pattern length. Most bending occurred in the 2 mm pattern according to the pattern length, and the smallest deformation occurred in the 1 mm pattern.

**Figure 14:**
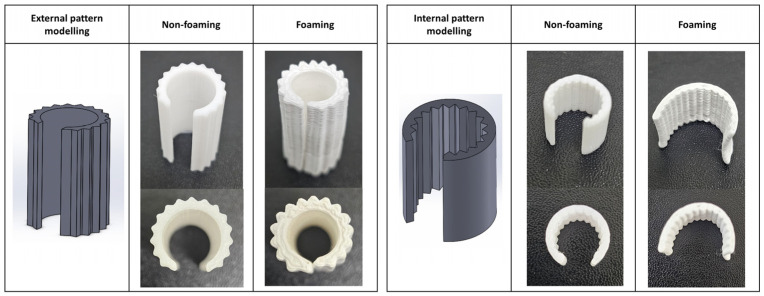
Application specimen of the external and internal pattern models.

As shown in Figure 14, the specimens with external patterns were fabricated and subjected to foaming. When foaming was applied to the externally patterned specimens, bending occurred inward towards the non-patterned side. This finding suggests potential future applications in developing medical devices, such as bandages, that provide structural support. Additionally, Figure 14 shows that when the foaming process was applied to the internally patterned specimens, bending occurred outward away from the patternless side.

## 4. Conclusions

This study proposes a new 4D printing process combining 3D printing and the MCP. By applying a foaming process to a patterned specimen manufactured using 3D printing, an efficient 4D printing motion was implemented. When the foaming process was applied, volume expansion occurred in the specimen, and asymmetric volume expansion occurred within the patterned part, resulting in bending. Existing 4D printing implements motion using smart materials. However, in this study, we used a common material, which is more cost-effective.

The degree of bending could be adjusted according to the patterning length and gas saturation time. When the patterning length was 2 mm, the largest self-deformation occurred, and the smallest self-deformation occurred in the 1 mm pattern. In addition, the cell morphology differed according to the gas saturation time. When the saturation time was less than 120 min, a heterogeneous cell was created, resulting in significant bending. After the saturation time of the gas reached 180 min, a homogeneous cell was formed, and the foaming rate decreased; that is, the amount of deformation decreased. In addition, when a homogeneous cell was generated, bending occurred proportionately to the surface area ratio. At 300 min, similar bending angles were obtained in the 1, 2, and 3 mm patterns with the same surface area ratio.

The proposed method provides a potentially more useful and efficient method for 4D printing compared to conventional approaches by using PLA as a single material, which is also a common material. In addition, microcellular foaming is an eco-friendly method that can reduce material usage. When used with environmentally friendly materials such as PLA, a new sustainable manufacturing technique can be introduced. This microcellular foaming 4D printing process can be further developed and used in various fields, such as soft robotics and medical equipment.

## Figures and Tables

**Table 1:** Experimental parameters for 3D printing.

Property	Value
Nozzle temperature (°C)	210
Bed temperature (°C)	60
Nozzle size (mm)	0.2
Layer thickness (mm)	0.2
Infill (%)	100
Infill pattern	Lines

**Table 2:** Experimental parameters of the MCP.

Property	Value
Saturation pressure (MPa)	5
Saturation temperature (°C)	20
Saturation time (min)	15, 30, 60, 120, 180, 300
Foaming temperature (°C)	110
Foaming time (s)	40

## Data Availability

Data are contained within the article.
